# Tick Paralysis in Spectacled Flying-Foxes (*Pteropus conspicillatus*) in North Queensland, Australia: Impact of a Ground-Dwelling Ectoparasite Finding an Arboreal Host

**DOI:** 10.1371/journal.pone.0073078

**Published:** 2013-09-16

**Authors:** Petra G. Buettner, David A. Westcott, Jennefer Maclean, Lawrence Brown, Adam McKeown, Ashleigh Johnson, Karen Wilson, David Blair, Jonathan Luly, Lee Skerratt, Reinhold Muller, Richard Speare

**Affiliations:** 1 School of Public Health, Tropical Medicine and Rehabilitation Sciences, James Cook University, Townsville, Australia; 2 CSIRO Ecosystem Sciences, Atherton, Queensland, Australia; 3 Centre for Tropical Environmental and Sustainability Science, School of Earth and Environmental Sciences and School of Marine and Tropical Biology, James Cook University, Cairns, Australia; 4 Tolga Bat Hospital, Atherton, Australia; 5 School of Marine and Tropical Biology, James Cook University, Townsville, Australia; 6 School of Earth and Environmental Sciences, James Cook University, Townsville, Australia; 7 Tropical Health Solutions Pty Ltd, Idalia, Townsville, Australia; University of Minnesota, United States of America

## Abstract

When a parasite finds a new wildlife host, impacts can be significant. In the late 1980s populations of Spectacled Flying-foxes (SFF) (*Pteropus conspicillatus*), a species confined, in Australia, to north Queensland became infected by paralysis tick (*Ixodes holocyclus*), resulting in mortality. This *Pteropus*-tick relationship was new to Australia. Curiously, the relationship was confined to several camps on the Atherton Tableland, north Queensland. It was hypothesised that an introduced plant, wild tobacco (*Solanum mauritianum*), had facilitated this new host-tick interaction. This study quantifies the impact of tick paralysis on SFF and investigates the relationship with climate. Retrospective analysis was carried out on records from the Tolga Bat Hospital for 1998–2010. Juvenile mortality rates were correlated to climate data using vector auto-regression. Mortality rates due to tick paralysis ranged between 11.6 per 10,000 bats in 2003 and 102.5 in 2009; more female than male adult bats were affected. Juvenile mortality rates were negatively correlated with the total rainfall in January to March and July to September of the same year while a positive correlation of these quarterly total rainfalls existed with the total population. All tick affected camps of SFF were located in the 80% core range of *S. mauritianum*. This initial analysis justifies further exploration of how an exotic plant might alter the relationship between a formerly ground-dwelling parasite and an arboreal host.

## Introduction

Disease is increasingly recognised as a factor in the population dynamics of wild animals. It has been shown to have caused catastrophic population crashes [1], [2], [3], is suggested to underpin regular population cycling [4], [5], and is hypothesised to interact in a complex manner with other determinants of population regulation such as predation [6], [7]. Disease has a major influence on individual life expectancy [8] as well as mating and reproductive success [8], [9]. Although wildlife disease is of particular interest when there is the potential for human or agricultural impact (i.e., zoonoses including emerging infectious diseases), it is also of concern in the context of the conservation management of threatened species [10], [11], [12]. Disease has been noted as a threatening process for, among others, amphibians, seals, whales, canids, birds, dasyurids and flying-foxes [12]–[19]. Pathogens that infect a new species of host can have marked effects on that species, the most spectacular example being the global pandemic due to the chytrid fungus (*Batrachochytrium dendrobatidis*) in amphibians resulting in massive mortalities with some species driven to extinction [15].

**Figure 1:**
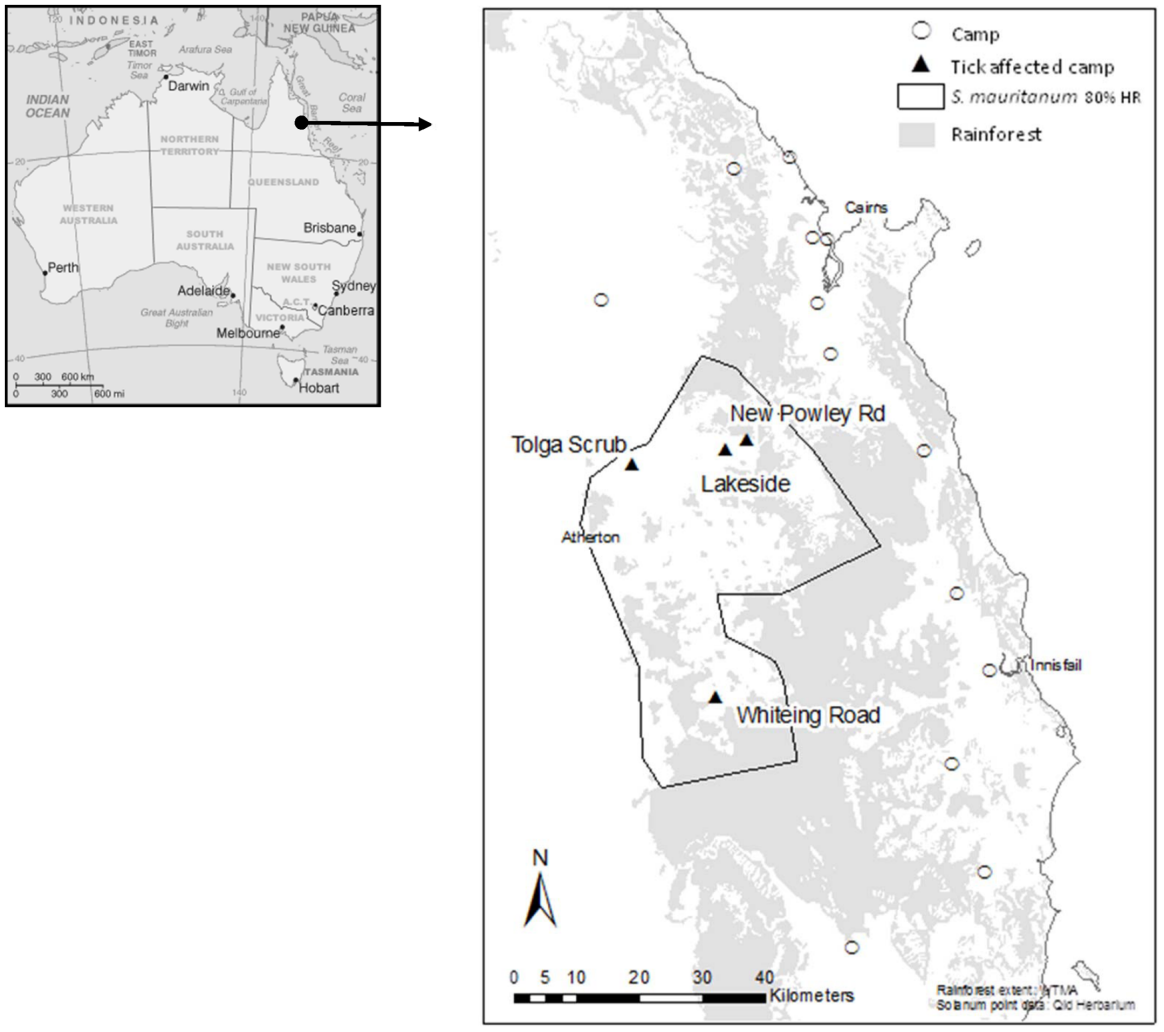
Distribution of spectacled flying-fox camps in the central Wet Tropics region, Atherton Tablelands, North Queensland, Australia. The distribution of rainforest is shown in grey. Enclosed area is the core distribution of *Solanum mauritianum* based on Queensland herbarium records for the Atherton Tableland (Source: Wikipedia; http://en.wikipedia.org/wiki/Australia; commons map; and CSIRO, Australia).

Spectacled flying-foxes (*Pteropus conspicillatus*; hereafter SFF) are primarily confined, in Australia, to the Wet Tropics region of North Queensland (Figure 1). They are effective pollinators and seed dispersers for sclerophyll and rainforest trees and provide unique seed dispersal services to a broad variety of plants [20], [21], [22] and micro-organisms [23]. The SFF was included in the Australian *Environment Protection and Biodiversity Conservation Act 1999* list of vulnerable species in May 2002 [24]. While the population trends of the species are unknown [25], SFF populations are thought to be threatened by a range of factors, including loss of habitat, electrocution on power lines, entrapment in fruit netting, shooting in orchards, entanglement on barbed wire, and most recently by tick paralysis [13], [20].

Australia has two species of paralysis tick, *Ixodes holocyclus* and *Ixodes cornuatus*, both found only in eastern Australia [26]. Although only the former occurs in northern Queensland, *I. holoyclus* is a species complex with 14 haplotypes found to date on the Atherton Tableland [27]. The Atherton Tableland is part of the Great Dividing Range of northern Australia; it is located south-west inland of Cairns (16.92° S, 145.78° E) and ranges in altitude between 500 m and 1250 m (Figure 1). *Ixodes holocyclus* is a three host tick: each stage, larva, nymph and adult female, feeds off a different individual host with a life cycle that can take from six months to a year [28], [29]. Although the different stages show some seasonality, adult females are present year round, but with higher numbers in spring and summer in southeast Queensland where *I. holocyclus* has been found on the lower foliage of shrubs and small trees [30]. Although bandicoots are the most important natural host of *I. holocyclus* [28], [30], [31], the tick will feed on a wide range of other hosts, including other wild and domestic mammals [32], and occasionally humans [33].

The toxin (actually a mix of proteins called holocytotoxin 1, 2 and 3) of *I. holocyclus* causes an ascending motor paralysis, combined with acute left-sided congestive heart failure, often resulting in death of the host [32]. Natural hosts typically develop immunity to the toxin through neutralising antibodies, but accidental hosts usually suffer morbidity and death. All tick stages are toxic [31], but clinical cases are usually produced by adult females, and rarely by larvae. Peak secretion of the toxin in the saliva of the tick is delayed until some days after attachment: adult female ticks reaching a maximum toxin secretion at the time of engorgement, approximately 4–5 days after attachment [32], [34].

The first recorded cases of tick paralysis in SFF caused by *I. holocyclus* occurred in the late 1980s when Bruce and Ann Johnson discovered 488 paralyzed flying-foxes in a colony at Zillie Falls near Millaa Millaa, Atherton Tableland [35]. Since then tick paralysis has been recorded in other camps on the Tablelands, but there has been no obvious sign of it in the coastal camps which typically contain the majority of the SFF population (A. McKeown, personal communication). It is important to note that *I. holocyclus* occurs on the coast in north Queensland, as well as on the Atherton Tablelands. Why this relationship has developed between SFF, an arboreal host, and *I. holocyclus*, considered a ground-dwelling ectoparasite, in only a portion of the overlapping ranges of both species is a perplexing question.

A number of factors might be associated with the epidemiology of tick paralysis in SFF. The ticks which infect SFF could be a specific genetic clade with unusual host preferences. Weather extremes could affect the distribution of SFF, their food sources, and patterns of activity in *I. holocyclus*. Other factors could also affect SFF food sources. SFF have started foraging in low-growing invasive tobacco bush (*Solanum mauritianum*) [23], [36]. Wild tobacco grows up to four metres tall and carries round yellow berries. It is native to South America but now invades disturbed sites throughout Queensland and New South Wales [37], [38]. Some researchers have suggested SFF feed on wild tobacco owing to a scarcity of native food due to the clearing of native Australian vegetation and increasing fragmentation of the landscape on the Atherton Tableland [23], [39]. The possibility that SFF choose wild tobacco as a food source over available native vegetation (rather than as a second class option) must also be considered. Native animals adapt to new environments and some select introduced plants as their preferred food [40]. In 2001, an unpublished collaborative study between the Tolga Bat Hospital and the Commonwealth Scientific and Industrial Research Organisation (CSIRO) in Atherton found *S. mauritianum* harbours more ticks than the native Australian food trees usually visited by SFF (J. Maclean, personal communication). The potential role of *S. mauritianum* in the relationship of SFF and *I. holocyclus* is poorly understood.

While tick paralysis kills individuals [41], the impact on the SFF population is unknown. Given that this species is currently listed as threatened there is a management imperative to understand its population dynamics. The only data available on the frequency of tick-induced morbidity and mortality in SFF comes from the privately funded Tolga Bat Hospital, a flying-fox rescue organisation located close to Atherton [42].

The paper (a) describes basic demographic characteristics of SFF affected by *I. holocyclus* between 1998 and 2010 on the Atherton Tableland; (b) estimates tick-related mortality of SFF; and (c) investigates whether climatic conditions are correlated with tick-related SFF mortality. The overlapping range of the core distribution of *S. mauritianum* and tick affected SFF camps is discussed.

## Methods

### Flying-fox population counts

During the study period SFF population counts were conducted by several different groups using two different monitoring methodologies: camp fly-out counts and day counts of roosting SFF. Between 1998 and 2003, and again in 2005, fly-out counts were conducted by the Queensland Parks and Wildlife Service in November or December of each year. The animals were counted over a period of three consecutive nights as they left the camp at dusk to forage [43]. Censuses were conducted for all known camps between the Daintree and Russell Rivers in 1998 [43]. From 1999 through 2003 and in 2005 the census included all known camps in the species' Wet Tropics distribution [44]–[49] (Figure 1). The counts included adult bats and juveniles surviving from previous breeding seasons. Young of the year are still attached to their mothers for about one month post-partum and are left in the camp thereafter and were therefore not counted. The numbers of camps surveyed per year varied between 9 and 33; in the mid 1990s not all camps were as yet known. In November 2009 an additional fly-out count was conducted solely at the Lakeside colony – the only known camp significantly affected by tick envenomation in that year.

From 2004 onwards day count population censuses that covered all known SFF camps in the species' Wet Tropics range have been conducted monthly by CSIRO ([50]; D. Westcott, unpublished data). During the day counts, transects were walked through the camp and the number of SFF per observed tree was estimated, along with a count of the number of trees. These estimates were then extrapolated to the entire camp based on an estimation of its areal extent [50]. In addition to population size, the monthly visits to all known camps allow the differentiation between tick affected and non-affected camps.

The use of two census methodologies might introduce some information bias into the population estimates used in this analysis. However, both methods result in an under-count of roughly the same magnitude, though stemming from different error sources [25], [51].

### Tick-related mortality

The Tolga Bat Hospital's [42] records of SFF affected by tick paralysis between 1998 and 2010 were retrospectively analysed. The bats included in those records were collected from four major SFF camps on the Atherton Tableland: Tolga Scrub (145°28.8′E, 17°13.86′S); New Powley Road (145°37.066′E, 17°12.187′S); Whiteing Road (145°36.3′E, 17°33.36′S); and Lakeside (145° 35.645′E, 17° 16.004′S) (Figure 1).

Data were collected by staff and volunteers of the Tolga Bat Hospital between September and January each year between 1998 and 2010 during their daily searches for ailing flying foxes. The number of dead animals and the number of animals affected by tick paralysis, stratified by age (adult and juvenile) and sex, were recorded daily. Dead adult animals for which sex could no longer be determined because of the advanced stage of decomposition (number between 3 and 59 SFF per year) were added to each gender count according to the known sex ratio of affected bats. The assumption is made that all orphaned juvenile SFF were coming into care because their mother had died of tick paralysis.

Tick envenomation was assigned as the cause of death when a tick was found in situ on a dead bat. For live bats, tick paralysis was diagnosed when a tick was found and clinical signs included a progressively worsening paralysis accompanied by respiratory distress. Tick envenomation was also assigned as the cause of death when no tick was present but the dead or ill bat exhibited signs consistent with tick envenomation, including a “tick crater” (a small ulcer with a surrounding inflamed, elevated area) on a part of the body where ticks are commonly encountered. Ill bats that had ticks but survived due to treatment were classed as “dead” for the present analysis since only human intervention saved their lives.

In 2006 only, the main bat colony affected by paralysis tick relocated from the Tolga Scrub to a new as yet unknown camp; hence, data for that year are missing. It is of interest to note that tropical cyclone Larry had a severe impact on the Atherton Tablelands in March 2006. For the years 2007 to 2009 no direct counts of tick paralysed adult bats were possible and in 2009 all bat searches had to stop for three weeks in November because too many juveniles were in care. For these years the number of affected adult animals was estimated based proportionally on the number of juveniles from previous years using linear regression (adults  = 83.66+1.075 x juvenile; Pearson's correlation coefficient  = 0.74; p = 0.035). For the 3 weeks in November 2009 when all searches were suspended, the number of affected juveniles was estimated based on the previously observed monthly distribution: 70.8% (range 57.4% to 83.8%) of all juveniles found between 2001 and 2008 and in 2010 were found in November of the respective year.

Mortality rates were calculated per 10,000 SFF for all affected animals combined and separately for female and male SFF using the overall population numbers provided by fly-out counts and day counts, as available. Mortality rates were calculated using both the original data (omitting the missing years) and the data with the extrapolated values for the missing years, and based on the counts of the total known SFF population as well as the population counts for the tick affected camps only. Spearman's correlation coefficient was used to assess time trends in the number of juvenile SFF affected by tick paralysis, as well as in the mortality rates. Counts of affected female adult and male adult SFF were compared using Wilcoxon signed rank test.

### Correlation with climate data

Median and total rainfall per month, percent of rainy days per month, mean maximum, mean minimum and mean temperature at 9 am per month, mean humidity, pan evaporation and hours of sunshine per month were all calculated based on daily data available from the Australian Bureau of Meteorology for the Kairi Research Station on the Atherton Tableland for 1998 through 2010 [52]. The monthly climate data were summed and/or averaged to create quarterly (Q1: Jan-Mar; Q2: Apr-Jun; Q3: Jul-Sep; Q4: Oct-Dec) measures.

The analysis of the correlation between tick disease and climate was limited to the data for orphaned juvenile bats, as these data were the most extensive (13 years) and complete (data missing for 2006 and November 2009). Data for 2006 were interpolated using the available monthly data: estimates of the rate of tick disease among juvenile bats for September, October, November and December were linearly interpolated based on the data for the corresponding months in previous and later years; these interpolated monthly estimates were then summed to create an estimate of the annual rate of juvenile tick disease for that year. We did not explore the relationship between climate and adult bat tick disease, but we believe the juvenile data are adequately representative of the colony. Using the nine years (1998–2005) of annual data with complete adult and juvenile counts; regression with Newey-West standard errors accounting for potential auto-correlation in time series data demonstrated significant correlation between juvenile tick paralysis rates and both adult female (*β* = 0.93; 95%CI: 0.78–1.08) and adult male (*β* = 0.69; 95%CI: 0.30–1.07) rates of tick paralysis.

We used vector auto-regression (VAR) to model the relationship between climate data and tick paralysis. VAR allows the inclusion of both endogenous variables (that is, dependent variables that might be affected by their own prior year values as well as by each other through some dynamic feedback mechanism) and exogenous variables (that is, independent variables that might affect the dependent variables, but are not themselves affected by any feedback mechanism from the dependent variables) [53]. We included the annual prevalence of juvenile SFF tick paralysis per 10,000 population in the affected camps as well as the population of the affected camps as endogenous variables; the climate variables were included as exogenous variables.

The Augmented Dickey-Fuller test was used to test for unit roots in both the endogenous and exogenous variables. Only those climate variables that lacked a unit root were included in the analysis. To minimise co-linearity (which is prohibited in VAR), the relationship between climate data variables that lacked a unit root and the endogenous variables was modelled first for each set of quarterly climate variables separately (that is, Q1 climate data and endogenous variables, then Q2 climate data and endogenous variables, and so on). Then, all of the significant quarterly climate variables were incorporated into one final model. The VAR model included two lagged values of the exogenous variables: the year prior and two years prior values (more lags introduced co-linearity).

Lastly, we used the final VAR to test for Granger causality between the endogenous variables using the Wald test, and to decompose the model forecast error variance. For all testing, an alpha value of 0.05 was used to establish statistical significance. The climate related analyses were performed using STATA, version 11 and PASW (SPSS version 18; IBM SPSS Inc; Chicago, Illinois).

### Ethics

No permits or ethical approvals were required. The study is purely descriptive analysing data retrospectively. Correlations with climate occurred on an ecological level. The study complied with all relevant regulations.

## Results

Total population estimates, as well as population estimates for the affected camps, varied substantially from year to year: the total population ranged between 74,400 (1999) and 269,728 (2005); the population of the affected camps ranged between 4,500 (2001) and 40,000 (2008). The number of animals found to be affected by *I. holocyclus* also varied greatly, from 165 in 1999 to 720 in the partial count of 2009 (Table 1). When extrapolating data from prior years to fill in the missing 2009 data, the estimate of tick-affected bats for that year increases to almost 1600 animals.

**Table 1:** Numbers of spectacled flying-foxes (*Pteropus conspicillatus*) affected by tick paralysis between 1998 and 2010 and population counts of *P. conspicillatus*.

	Year												
	1998	1999	2000	2001	2002	2003	2004	2005	2006^$^	2007^$$^	2008^$$^	2009^#^	2010
Total Camp Population (x 1,000) estimates for November**													
Fly-Out	113.96	74.40	79.98	187.18	194.90	172.75	/	269.73	/	/	/	*/*	*/*
Counts													
Day	/	/	/	/	/	/	250.27	214.75	152.70	137.00	159.00	154.00	100.52
Counts													
Affected Camp Population (x 1,000) estimates for November**													
Fly-Out	18.00	5.50	12.00	4.50	49.06	22.34	/	33.72	/	/	/	27.20	/
Counts													
Day	/	/	/	/	/	/	23.00	35.00	10.00	25.00	40.00	30.00	30.00
Counts													
**Total number of affected animals (dead or alive)** *													
Adult	166	52	289	220	152	78	227	199	/	627	533	858	107
Female													
Adult	88	21	97	200	149	53	51	65	/	/	/	/	30
Male													
Juvenile	114	92	297	208	124	70	184	249	23	505	418	720	115

* Affected animals from Tolga scrub, Whiteing Road, and New Powley Road on the Atherton Tableland, North Queensland, Australia; **2006 count is taken from December and includes Lakeside a new camp affected by tick paralysis; ^$^Animals moved to new unknown camp (Lakeside) so searches were incomplete in 2006; ^$$^No details about numbers of dead adult animals available, as too many juvenile animals were in care; number of adult *P. conspicillatus* given were extrapolated from previous data; **^#^**Search for affected animals stopped for 3 weeks because too many juveniles were in care; number of adult and juvenile animals based on seasonal distribution of previous years.

For the years with complete counts (1998 to 2005; 2010) more female adults than male adults were affected by tick paralysis (p = 0.008). This finding remains unchanged when dead animals for which gender could no longer be obtained were excluded. The number of juvenile SFF in care because of tick paralysis increased over the 13 year observation period by an average 28 animals per year, but this trend was not statistically significant (Spearman's r = 0.50; p = 0.101).

Mortality rates of the total SFF population caused by tick paralysis ranged between 11.6 per 10,000 SFF in 2003 and 85 per 10,000 in 2000 (Table 2). When mortality rates were extrapolated from previous distributions for years with incomplete data, the highest estimate was 102.5 per 10,000 SFF for 2009 (Table 2). Tick-related mortality rates trended downward year over year when basing the analysis on the years with complete data and the population of the affected camps (Spearman's r = −0.68; p = 0.042). There was no correlation between time and tick-related mortality when the analysis was based on the years with complete data and the total population (Spearman's r = −0.52; p = 0.154), or when including the extrapolated data for 2007 to 2009 (total population mortality: Spearman's r = 0.16; p = 0.618; affected camps mortality: Spearman's r = −0.25; p = 0.430).

**Table 2:** Mortality rates (per 10,000) of spectacled flying-foxes (*Pteropus conspicillatus*) caused by tick paralysis on the Atherton Tableland, North Queensland, Australia.

	Year												
	1998	1999	2000	2001	2002	2003	2004	2005	2006^$^	2007^$$^	2008^$$^	2009^#^	2010
**Based on total population fly-out counts and [** ***day counts*** **].** *													
All	32.3	22.2	85.4	33.6	21.8	11.6	NA	19.0	/	NA	NA	NA	NA
Affected	[NA**]	[*NA*]	[*NA*]	[*NA*]	[*NA*]	[*NA*]	[*18.5*]	[*23.9*]	/	[*82.6*]	[*59.8*]	[*102.5*]	[*25.1*]
Adult	14.6	7.0	36.1	11.8	7.8	4.5	NA	7.4	/	/	/	/	NA
Female	[*NA*]	[*NA*]	[*NA*]	[*NA*]	[*NA*]	[*NA*]	[*9.1*]	[*9.3*]	/	/	/	/	[*10.6*]
Adult	7.7	2.8	12.1	10.7	7.6	3.1	NA	2.4	/	/	/	/	NA
Male	[*NA*]	[*NA*]	[*NA*]	[*NA*]	[*NA*]	[*NA*]	[*2.0*]	[*3.0*]	/	/	/	/	[*3.0*]
Juvenile	10.0	12.4	37.1	11.1	6.4	4.1	NA	9.2	/	NA	NA	NA	NA
	[*NA*]	[*NA*]	[*NA*]	[*NA*]	[*NA*]	[*NA*]	[*7.4*]	[*11.6*]	/	[*36.9*]	[*26.3*]	[*46.8*]	[*11.4*]
**Based on affected camp population fly-out counts and [** ***day counts*** **].** *													
All	204.4	300.0	569.2	1395.6	86.6	90.0	NA	152.1	/	NA	NA	580.1	NA
Affected	[*NA*]	[*NA*]	[*NA*]	[*NA*]	[*NA*]	[*NA*]	[*200.9*]	[*146.6*]	/	[*452.6*]	[*237.8*]	[*526.0*]	[*84.0*]
Adult	92.2	94.6	240.8	488.9	31.0	34.9	NA	59.0	/	/	/	/	NA
Females	[*NA*]	[*NA*]	[*NA*]	[*NA*]	[*NA*]	[*NA*]	[*98.7*]	[*56.9*]	/	/	/	/	[*35.7*]
Adult	48.9	38.2	80.8	444.4	30.4	23.7	NA	19.3	/	/	/	/	NA
Males	[*NA*]	[*NA*]	[*NA*]	[*NA*]	[*NA*]	[*NA*]	[*22.2*]	[*18.6*]	/	/	/	/	[*10.0*]
Juvenile	63.3	167.3	247.5	462.2	25.3	31.3	NA	73.9	/	NA	NA	264.7	NA
	[*NA*]	[*NA*]	[*NA*]	[*NA*]	[*NA*]	[*NA*]	[*80.0*]	[*71.1*]	/	[*202.0*]	[*104.5*]	[*240.0*]	[*38.3*]

* Upper mortality estimate is based on fly-out counts, lower number is based on day counts as available; **NA  =  not available; ^$^Animals moved to new unknown camp (Lakeside) so searches were incomplete in 2006; ^$$^No details about numbers of dead adult animals available, as too many juvenile animals were in care; numbers given were extrapolated from previous years.

### Climatic conditions and tick paralysis in juvenile SFF at affected camps

**Figure 2:**
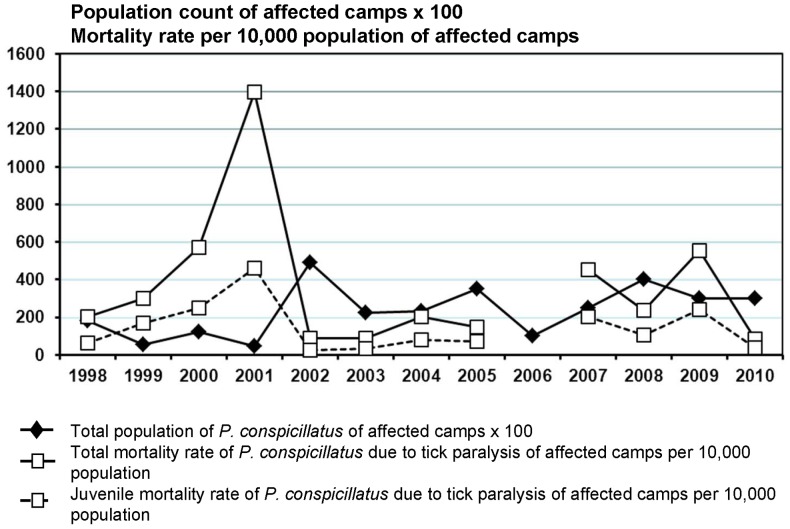
Juvenile *Pteropus conspicillatus* tick paralysis rate per 10,000 population per year, and total *P. conspicillatus* tick paralysis rate per 10,000 population per year in the affected camps as well as total population of affected camps over the 13 year study period.

Figure 2 shows the rate of juvenile SFF tick paralysis per 10,000 population in the affected camps over the 13 years, along with the population of the affected camps. There are no obvious trends or cyclical patterns over time, but the total population does appear to shadow the rate of juvenile tick disease; this is most apparent for the anomalous peaks in 2001 and 2002.

Although there was weak evidence of a unit root (ADF  = −2.743; p = 0.069) in the rate of juvenile tick paralysis, this was not sustained when including a drift term in the regression. There is also a theoretical basis for rejecting a unit root in these data: the disease rate is absolutely bounded in that it cannot go below ‘0’ and there is an (unknown) maximum disease rate above which the colony could not survive. There was no evidence of a unit root in the population of the affected camps (ADF  = −3.129, p = 0.023). Table 3 lists those quarterly climate variables for which we could also reject the presence of a unit root. Although including a drift term in the regression overcame the unit root for many of the other climate variables, they inevitably exhibited co-linearity with the variables listed in Table 3 and were therefore not included in the climate analysis.

**Table 3:** Climate variables without a unit root in the yearly quarters (Q).

Q1: Jan-Mar	Q2: Apr-Jun	Q3: Jul-Sep	Q4: Oct-Dec
Total Rainfall	Total Rainfall	Total Rainfall	Total Rainfall
Max Temperature	Max Temperature	Humidity	9am Temperature
9am Temperature	9am Temperature	Hours of Sunshine	Min Temperature
	Humidity	Pan Evaporation	
	Hours of Sunshine		

Max  =  maximum; Min  =  minimum.

There were no significant associations between Q2 (Apr-Jun) climate variables and the endogenous variables; conversely, all of the Q3 (Jul-Sep) climate variables significantly contributed to the modelling. Only quarterly total rainfall (except for Q2) consistently contributed to the modelling (data not shown). Those quarterly climate variables that were significantly associated with either the rate of tick disease in juvenile bats or the population of the affected camps were retained in the final analysis and are shown in Table 4, along with the results of the final VAR.

**Table 4:** Final vector auto-regression (VAR) model predicting mortality rates of spectacled flying-foxes (*Pteropus conspicillatus*) caused by tick paralysis and total population count of affected camps.

Dependent Variable	Independent Variables	Coefficient	95% Confidence interval	p-value
Annual prevalence	L1 Disease Rate	−0.561	−1.139; 0.017	0.057
	L2 Disease Rate	−1.352	−2.235; −0.468	0.003
	L1 Population	−0.0002	−0.007; 0.007	0.959
	L2 Population	0.0107	−0.015; −0.006	<0.001
	Q1 Total Rainfall	−0.218	−0.406; −0.030	0.023
	Q3 Total Rainfall	−02.792	−4.254; −1.330	<0.001
	Q4 Total Rainfall	0.122	−0.134; 0.378	0.350
	Q4 9am Temperature	35.357	−25.403; 96.118	0.254
Population of affected camps	L1 Disease Rate	110.977	49.597; 172.357	<0.001
	L2 Disease Rate	113.004	19.186; 206.8.22	0.018
	L1 Population	−0.025	−0.772; 0.721	0.947
	L2 Population	1.035	0.544; 1.526	<0.001
	Q1 Total Rainfall	25.606	5.632; 45.581	0.012
	Q3 Total Rainfall	248.009	92.777; 403.241	0.002
	Q4 Total Rainfall	−5.484	−32.678; 21.711	0.693
	Q4 9am Temperature	2693.723	−3758.347; 9145.793	0.413

L1  =  one year prior; L2 =  two years prior. Variables omitted from the analysis due to co-linearity include: Q1 Maximum Temperature; Q3 Humidity; Q3 Hours of Sunshine; Q3 Evaporation; Q4 Minimum Temperature.

There were significant negative associations between Q1 and Q3 rainfall and the rate of juvenile tick paralysis, and significant positive associations between Q1 and Q3 rainfall and the population of the affected camps. Adding the ‘year’ into the model to introduce a potential time-related effect did not meaningfully alter the size, sign, or significance of any of the coefficients, and ‘year’ itself did not significantly contribute to the model.

Granger causality testing confirmed the feedback loop between the rate of juvenile tick paralysis and the population of the affected camps; the rate of juvenile tick paralysis Granger-causes the population of affected camps (X^2^  = 26.591, p<0.001) and the population of the affected camps Granger-causes the rate of juvenile tick disease (X^2^  = 34.863, p<0.001). Only the influence of the rate of juvenile tick disease on the population of affected camps, however, is apparent in the impulse response functions and the decomposition of the forecast error variance. When modelling the population of the affected camps for two or three years into the future, 70% to 80% of the forecast error variance is explained by innovations in the rate of juvenile tick paralysis. To the contrary, when modelling the rate of juvenile tick paralysis, only small portions (24% to 33%) of the forecast error variance can be explained by innovations in the population of the affected camps, and the 95% confidence intervals around the explainable portions of those forecast error variances all include ‘0’ (data not shown).

## Discussion

This is the first report to quantify the impact of tick paralysis on any population of flying foxes globally. Quantification is a challenge since the denominator changes frequently as SFF move between camps, both upland and lowland, in a seemingly unpredictable manner. The analysis found that the raw numbers of SFF affected by *I. holocyclus* rose markedly between 1998 and 2010, although mortality rates remained statistically stable over the study period since numbers of observed SFF (the denominator) in the studied camps also increased. This study also found an association between climate patterns and the rate of tick paralysis in juvenile SFF.

Mortality rates were stable over the course of our study, although the numbers of affected SFF increased markedly. This was due to an increase in observed SFF during the tick season, but it is unclear whether this increase was due to more individuals or a greater proportion of the population being observed. The numbers of affected SFF detected in camps varied substantially by year. When the censuses of SFF first began, counts increased in successive years as more camps were detected [54]. Other papers suggest that the SFF population on the Atherton Tableland is in decline [39]. Despite evidence of potential ages in the wild in excess of 13 years, Fox et al (2008) found 93% of their SFF sample of tick-affected animals were 6 years or less in age and estimated the annual mortality rate of SFF to be about 35% [39]. The present data suggests that tick paralysis contributes at least 1% to this overall mortality rate. Whether this level of tick-induced mortality is significant, however, cannot currently be determined due to the considerable inter-annual variation in estimated population size.

Consistent with the visual impression given by Figure 2, our data suggest that population levels in the affected camps are foreshadowed by prior year(s) levels of juvenile tick paralysis rates, although the VAR and Granger causality testing do demonstrate that each endogenous variable has some influence on the other. This observation cannot be explained by reproduction but may add weight to the “population sink hypothesis”. High gene flow between SFF camps, inferred using six microsatellite loci, suggests that the species forms one large breeding population scattered throughout the Wet Tropics of Australia [55], [56]. However, it would be interesting to investigate individual variation in susceptibility and whether selection for resistance is occurring. The SFF camps known to be affected by tick paralysis are all located at the southern end of the Atherton Tableland, suggesting these camps may be a population sink for bats moving to the Tableland from camps where *I. holocyclus* does not parasitise SFF [39]; bats that die from tick paralysis are possibly replaced by new arrivals.

We found higher mortality for adult female SFF compared to males in each of the eight years during which sex distribution was recorded. The reasons for this observation are unclear but may include (a) affected camps are predominantly maternity camps with few males present; (b) females with babies have different foraging behaviour than males, resulting in different exposure to ticks; and/or (c) males are potentially less likely to return to camps due to competition [57], thus dying away from the camps and not being included in the counts based on camp surveys. However, the loss of many female SFF of reproductive age is clearly detrimental for the species. The SFF has a generation length of approximately four years, low fecundity (one young per year), and an assumed high infant mortality rate [13], [39], [58], [59]. Although females become fertile in their second year, it is rare for them to successfully raise an offspring before they reach three years of age [59].

Wild tobacco (*S. mauritianum*) was first recorded on the Tableland in 1929 [60], and has been considered a major pastoral weed in the region ever since. It appears to favour disturbed areas at mid elevation and with higher rainfall. There are very few records from areas with a much drier climate or along the coastal strip. A map of the distribution of *S. mauritianum* was considered which was based on all available herbarium collection records from the Wet Tropics, Australia [60] and which provided the 80% core range for *S. mauritianum* [61] (Figure 1). It is an interesting observation that all of the SFF camps within this core range are tick-affected, whereas none of the camps outside this range are affected, thus providing some ecological evidence for a possible role of *S. mauritianum* in the relationship between SFF and paralysis ticks. SFF eat the berries of wild tobacco [23], [36] and might forage closer to the ground than usual, allowing for exposure to ticks. *Solanum mauritianum* might harbour more ticks than the native Australian trees usually visited by SFF. Introduced plants and plant pathogens may modify tick dynamics: exotic grasses in eastern USA increased the mortality in two species of ticks by increasing temperature and reducing humidity in soil microhabitats used by these species [62]; sudden oak death (due to an introduced fungal pathogen) in California decreased the incidence of carriage of the Lyme disease bacterium by nymphal ticks [63].

This study found that higher first and third quarterly total rainfalls were associated with lower rates of juvenile tick paralysis, an association which might be linked to tick abundance through multiple pathways. We did not find any impact of the second quarter rainfall, a time at which the larvae and nymphs should be equally vulnerable, but this could simply be an artefact of the consistently drier nature of the Atherton Tableland during that quarter: the average total rainfall for that quarter in our data was 175 mm, with the total exceeding 255 mm in only one of the 13 years. Alternatively the observed correlation between mortality rates from tick paralysis and climatic conditions could be due to food availability. Weather patterns dictate the flowering and fruiting of plants and this in turn will determine what SFF eat. Hence the weather pattern might indirectly influence whether and where SFF and *I. holocyclus* meet.

Since the free-living stages of ticks have very limited capacity for independent movement, hosts play the major role in the distribution of ticks through a region [64]. Although the role SFF play in distributing *I. holocyclus* in the Wet Tropics is unknown, of all potential wild hosts SFF have the greatest capacity to carry *I. holocyclus* large distances in short periods of time. If a bat acquired a tick in one site in the region, it could potentially move the tick hundreds of kilometres before becoming affected by the tick toxin 4 or 5 days later. In some species of ticks clades show subtle differences in adaptation to climatic variables, resulting in their occupation of different parts of the species range [64]. Since *I. holocyclus* has a large number of haplotypes [27], perhaps particular clades have adapted to utilizing SFF. Obtaining data on the stages and clades of *I. holocyclus* feeding on bats as well as the clades of ticks found free-living in various locations in far north Queensland would assist in clarifying this complex host-ectoparasite interaction.

This study also highlights that community-based organisations can play a valuable role in collecting data to help understand diseases of wildlife. In particular, it illustrates what an important role a community organisation, dedicated to a specific group of animals such as bats (i.e., Tolga Bat Hospital), can play in identifying possible disease risks that can threaten species recovery. Similar observations were made for data on amphibian diseases collected by the Cairns Frog Hospital; the data are imperfect, but the analysis can highlight particular diseases that need more rigorous study [65]. The contribution of such organisations towards an understanding of disease epidemiology and species ecology can be increased by collaborative activity with relevant researchers. This is a model which should receive greater consideration from research and policy funding bodies.

## Limitations

We acknowledge that the quality of data collected by volunteers of community organisations may vary and processes may lack the rigour of data collected by trained research personnel. In the present study, searches for tick affected animals and respective data collection were organised and supervised by J.M. and A.J. The Tolga Bat Hospital has a history of successful collaboration with research institutions and staff understand the importance of high quality data.

Our analysis was limited as no data for affected adult SFF were available for 2006 to 2009 and for juvenile SFF for 2006 and November 2009, requiring extrapolation of data based on previous years in order to complete the VAR analysis exploring the relationship between climate and juvenile tick paralysis. For our other results, we presented both the raw and extrapolated data. In either case, it can be assumed that the number of affected animals reported is an underestimation of true mortality figures. Previous studies have shown that SFF can fly at least 50 km in one night when searching for food [66]. SFF seem to encounter ticks when foraging and many SFF could be paralysed and die while away from their camp and thus never be identified as tick victims.

The present analysis assumed that observed death of SFF was caused by tick paralysis. Most adult SFF were found with ticks still attached to their body. Predation of bats would leave a mangled body. Flying foxes accounted for in this analysis had no obvious signs of predation. There is the possibility that ticks attach only after a bat has fallen dead to the ground. However, it is well established that ticks are attracted to living animals by CO_2_ [67]. Also, over the study period a minimum of two people were spending 1–2 hours every day for four months every year in the tick affected camps and never picked up one tick. Hence the camp grounds seem to be virtually tick free. In addition, in a related study a PhD student (K. Wilson) has been conducting post-mortems on more than 100 adult flying foxes which had died due to tick paralysis. These post-mortems found the animals free from other potentially fatal pathogens apart from one animal with Australian Bat Lyssavirus. Also, the majority of juvenile SFF were found on dead mothers and most of these mothers still had a tick attached. In the case of abandoned juveniles one cannot be 100% sure whether the adult mother is tick affected or not. However, abandoned juveniles made up less than 10% of all juveniles counted. In addition, abandoned juveniles are very rare in colonies without tick paralysis (A. McKeown, personal communication).

It is also very difficult to establish reliable population estimates for flying foxes because of the propensity for animals to change camps [20], [25], [66] and for large camps to disappear within days [50], [58], [68], [69], [70]. In the present analysis we used the results of two methods of establishing population data for flying foxes, fly-out counts and walk through day counts. Day counts avoid the errors of fly-out counts, such as missing a stream of flying out animals, but they are in general assumed to be less accurate due to reduced visibility of animals hanging closely together high up in trees and disturbance caused by the observer [25]. On the other hand, day counts have been found to have a higher level of precision ([71] cited in [25]). In addition, monthly censuses of known camps show extreme variations in size [50] and it has been suggested that a variable proportion of the population roosts away from known camps at any given point in time, creating an important source of error for population counts. In a study on observer error of fly-out counts Westcott and McKeown [51] compared fly-out counts with video counts of the same stream of animals, showing that fly-out counts under-estimate the video count by 14.7% (SD±25). Based on these observations we concluded that both methods are prone to an under-estimation of the flying-fox population. For 2005, total population counts were available by both methods, however varying by 25.6%. On the other hand, in 2005 two camps were tick affected and both were counted through fly-out and day count with the counts varying by only about 3.8%. Hence time trends of mortality rates based on population counts of affected camps should be reasonably reliable.

Estimates of mortality rates based on the total number of animals in affected camps are potentially further limited because camp censuses were usually conducted over a relatively short period of time, for example, during three consecutive days in November. Thus, the higher mortality rate in 2001 could be caused by an error in estimating the actual number of SFF in the affected camp [46]. On the other hand, female SFF give birth in November and this is not a time when substantial numbers of animals are likely to move between camps.

## Conclusions

This study has demonstrated that the paralysis tick (*I. hylocyclus*), usually considered a ground-dwelling ectoparasite, is affecting large numbers of SFF, an arboreal host, in only a portion of the host's range. The numbers of SFF in the study area, and the numbers and rates of SFF affected, were highly variable reflecting the difficulty of obtaining accurate population counts for a species that is highly mobile within its host range. More adult females were affected than adult males, and importantly higher juvenile mortality rates were observed during drier years. All of the tick-affected SFF camps lay within the core range of introduced wild tobacco, *S. mauritianum*. Further studies are needed to better understand if this introduced plant is the major risk factor for acquisition of paralysis tick by this vulnerable host species, whether specific clades of *I. holocyclus* are involved, and whether feasible strategies to decrease the impact of tick paralysis can be developed.
